# Applications of chitosan derivatives as adjuvants for nanoparticle‐based vaccines: A comprehensive review

**DOI:** 10.1002/smo2.70060

**Published:** 2026-06-17

**Authors:** Sayantani Dasgupta, Susanta Paul, Swarupananda Mukherjee, Nilanjan Sarkar, Arvind Kumar, Amrita Chakraborty, Anannya Bose, Atanu Hazra, Mainak Chakraborty

**Affiliations:** ^1^ Department of Pharmaceutical Technology NSHM Knowledge Campus Kolkata – Group of Institutions Kolkata West Bengal India; ^2^ Barasat Bidhan Chandra Roy Institute of Pharmacy Kolkata India; ^3^ Calcutta Institute of Pharmaceutical Technology & Allied Health Sciences Howrah West Bengal India; ^4^ Department of Pharmaceutical Technology Adamas University Kolkata West Bengal India

**Keywords:** adjuvant, chitosan derivatives, immune modulation, mucosal immunity, nanoparticles, vaccine delivery

## Abstract

The development of safe and effective vaccines remains a critical priority in modern medicine. Traditional adjuvants, while effective in enhancing immune responses, often suffer from limitations including local reactogenicity, limited mucosal immunity, and instability of antigens. Chitosan, a natural polysaccharide derived from chitin, and its derivatives have emerged as promising candidates for nanoparticle‐based vaccine delivery due to their biocompatibility, biodegradability, mucoadhesive properties, and intrinsic immunostimulatory activity. Chemical modifications of chitosan, such as trimethylation, quaternization, and thiolation, enhance its solubility, stability, and immune modulatory functions. Chitosan derivative nanoparticles (CDNPs) facilitate efficient antigen encapsulation, protect labile biomolecules, and promote uptake by antigen‐presenting cells. They can trigger both humoral and cellular immune responses, particularly through mucosal administration routes. This review comprehensively discusses the physicochemical properties of chitosan derivatives, mechanisms of immune enhancement, methods for nanoparticle preparation, applications in preclinical and clinical vaccine studies, and challenges for translation to human use. Insights from recent literature suggest that CDNPs hold significant potential to revolutionize vaccine delivery platforms, especially for mucosal vaccines and next‐generation immunotherapeutics.

## Graphical abstract

This review highlights how chitosan derivatives enhance nanoparticle‐based vaccines through superior stability, mucoadhesion, and immune modulation. By triggering robust mucosal, humoral, and cellular responses, these biocompatible polymers serve as a transformative platform for next‐generation immunotherapeutics and safe and effective vaccine delivery systems.

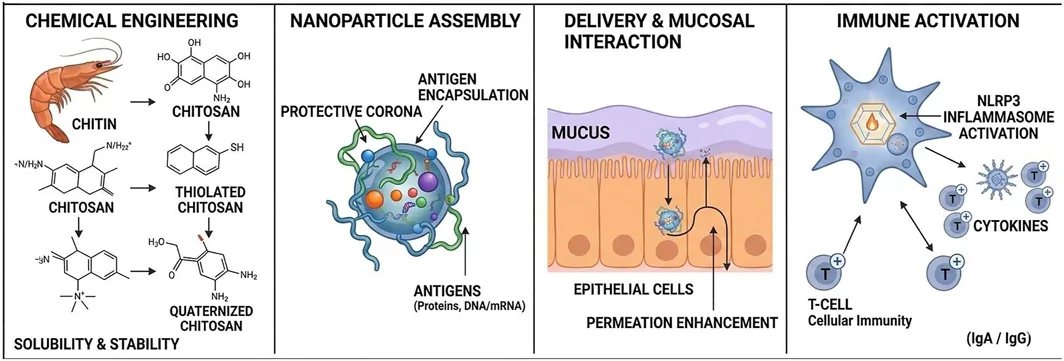

## 1 INTRODUCTION

Vaccines are one of the most effective strategies for preventing infectious diseases and controlling pandemics. However, the efficacy of modern vaccines often relies on the presence of adjuvants that enhance immunogenicity, stabilize antigens, and direct appropriate immune responses. Conventional adjuvants, such as aluminum salts and oil‐in‐water emulsions, have several limitations, including local inflammation, poor induction of mucosal immunity, and inability to deliver sensitive biomolecules like nucleic acids or proteins efficiently.[^1^, ^2^]

Chitosan, a linear polysaccharide obtained from deacetylation of chitin from crustacean shells, has garnered significant attention as a natural polymeric adjuvant and delivery vehicle. Its biodegradability, low toxicity, and inherent cationic nature allow it to interact with negatively charged biomolecules and cell membranes. Additionally, chitosan exhibits mucoadhesive properties, which are particularly advantageous for mucosal vaccine delivery, a route critical for combating pathogens that invade through respiratory or gastrointestinal tracts.[^3^, ^4^]

Chemical modification of chitosan enhances solubility at physiological pH and improves immunostimulatory activity. Derivatives such as *N*‐trimethyl chitosan (TMC), thiolated chitosan, and carboxymethyl chitosan (CMC) have shown superior antigen delivery and adjuvant functions compared to native chitosan.^[5]^ Encapsulation of antigens into chitosan derivative nanoparticles (CDNPs) protects labile molecules, improves uptake by dendritic cells and macrophages, and promotes controlled release. This review aims to provide a comprehensive and critical analysis of CDNPs, with particular emphasis on comparing different chitosan derivatives in terms of their physicochemical properties, immunological performance, and translational potential.

## 2 CHITOSAN DERIVATIVES: PROPERTIES AND MODIFICATIONS

### 2.1 Structural basis of chitosan derivatives

Chitosan is composed of *β*‐(1 → 4)‐linked D‐glucosamine and *N*‐acetyl‐D‐glucosamine units. Its cationic nature under acidic conditions enables interactions with negatively charged antigens, nucleic acids, and cell membranes. Solubility in aqueous media is limited at neutral and alkaline pH, prompting the development of water‐soluble derivatives for vaccine delivery applications.^[6]^

To establish a clear relationship between structural modification and functional performance, it is important to consider the core chemical structure of chitosan and its derivatives. Chitosan consists of *β* (1 → 4) linked D glucosamine units with primary amine groups at the C2 position and hydroxyl groups at the C3 and C6 positions, which serve as key sites for chemical modification. Structural derivatization of chitosan primarily involves substitution at these reactive functional groups, leading to significant changes in physicochemical and biological properties. For instance, quaternization of the amino group, as observed in TMC, introduces a permanent positive charge, resulting in enhanced solubility at physiological pH and improved interaction with negatively charged cell membranes, thereby increasing epithelial permeability and cellular uptake. In contrast, thiolation introduces sulfhydryl groups that enable the formation of disulfide bonds with mucin glycoproteins, significantly enhancing mucoadhesion and prolonging residence time at mucosal surfaces. Similarly, carboxymethylation introduces carboxyl groups that improve aqueous solubility and biocompatibility while reducing overall charge density, which influences antigen binding and release behavior. These structural modifications directly impact key performance parameters such as particle size, zeta potential, encapsulation efficiency, and immune activation. Therefore, understanding the relationship between chemical structure and functional properties is essential for the rational design and optimization of CDNPs for vaccine delivery applications.

### 2.2 Common derivatives

Several chitosan derivatives have been developed to overcome the limitations of native chitosan and enhance its suitability as a vaccine delivery vehicle. TMC is widely used due to its improved water solubility at neutral pH, which facilitates enhanced mucoadhesion and promotes paracellular transport of macromolecules across epithelial barriers. Quaternized chitosan carries permanent positive charges, which not only improve antigen binding but also increase the stability of the formulation under physiological conditions. Thiolated chitosan has been engineered to form disulfide bonds with mucosal surfaces, thereby prolonging retention time and allowing controlled release of encapsulated antigens. Additionally, CMC exhibits increased solubility and biocompatibility while preserving the intrinsic immunostimulatory properties of chitosan, making it a versatile candidate for nanoparticle‐based vaccine delivery.[^7^, ^8^, ^9^, ^10^]

### 2.3 Advantages over conventional adjuvants

Chitosan derivatives offer several advantages over conventional vaccine adjuvants, making them highly attractive for nanoparticle‐based delivery systems. They are inherently biodegradable and non‐toxic, ensuring safety for clinical applications. Their versatile structure allows them to encapsulate a wide range of antigens, including proteins, peptides, DNA, and RNA, thereby protecting labile biomolecules from degradation. Additionally, their mucoadhesive properties enhance retention at mucosal surfaces, promoting efficient antigen uptake and localized immune activation. Importantly, chitosan derivatives are capable of eliciting both humoral and cellular immune responses, including the production of mucosal IgA antibodies, which are essential for preventing pathogen entry at mucosal sites.[^11^, ^12^]

### 2.4 Comparative evaluation of major chitosan derivatives

While numerous chitosan derivatives have been explored for vaccine delivery, their performance varies significantly depending on physicochemical properties, degree of substitution, and formulation strategy. A critical comparison reveals distinct advantages and limitations among the commonly used derivatives (Table 1).

**TABLE 1:** Comparative evaluation of major chitosan derivatives used in nanoparticle‐based vaccine delivery systems.

Chitosan derivative	Particle size (nm)	Zeta potential (mV)	Encapsulation efficiency (%)	Key advantages	Limitations	Typical applications
TMC	150–300	+25 to +45	70–90	High solubility at physiological pH; enhanced epithelial permeability; strong mucoadhesion; improved antigen uptake	Potential cytotoxicity at high quaternization levels; charge‐dependent toxicity	Mucosal vaccines (intranasal, oral), protein and peptide delivery
Thiolated chitosan	180–350	+20 to +35	65–85	Strong mucoadhesion via disulfide bonding; prolonged residence time; enhanced transfection efficiency (2–3 fold)	Susceptible to oxidation; stability concerns during storage	DNA and RNA vaccines, mucosal delivery
CMC	200–400	−10 to +20	50–70	High water solubility; excellent biocompatibility; controlled and sustained release	Reduced cellular uptake due to lower positive charge	Sustained‐release systems, protein delivery
Quaternized chitosan	150–280	+30 to +50	75–90	Strong electrostatic interaction; high formulation stability; enhanced cellular internalization	Risk of non‐specific interactions; potential cytotoxicity at high charge density	Subunit vaccines, antigen delivery systems
Native chitosan	200–500	+15 to +30	40–70	Biodegradable; low toxicity; inherent adjuvant properties	Poor solubility at neutral pH; limited permeability	Conventional and experimental vaccine formulations

Abbreviations: CMC, carboxymethyl chitosan; TMC, *N*‐trimethyl chitosan.

TMC is one of the most extensively studied derivatives due to its permanent positive charge and excellent solubility at physiological pH. These properties enable enhanced epithelial permeability and efficient paracellular transport of antigens. Studies report that TMC nanoparticles typically exhibit particle sizes in the range of 150–300 nm, with encapsulation efficiencies often exceeding 70%–90%, making them highly effective for mucosal vaccine delivery. However, excessive quaternization may lead to increased cytotoxicity and reduced biocompatibility, highlighting the need for careful optimization.

Thiolated chitosan demonstrates superior mucoadhesive properties due to its ability to form disulfide bonds with mucin glycoproteins. This results in prolonged mucosal residence time and controlled antigen release. Thiolated chitosan‐based nanoparticles have shown enhanced cellular uptake and up to 2–3 fold higher transfection efficiency in nucleic acid delivery compared to native chitosan. Nevertheless, their stability can be affected by the oxidation of thiol groups, which may limit their long‐term storage.

CMC, in contrast, offers excellent aqueous solubility and improved biocompatibility with reduced cytotoxicity. Nanoparticles formulated with CMC generally exhibit moderate encapsulation efficiency (50%–70%) and more controlled sustained release profiles. However, the reduced cationic charge compared to TMC can result in weaker interactions with negatively charged antigens, potentially limiting cellular uptake efficiency.

Quaternized chitosan derivatives provide strong electrostatic interactions with antigens and improved formulation stability under physiological conditions. These systems have demonstrated high zeta potential values (+20 to +40 mV), contributing to enhanced cellular internalization. However, similar to TMC, excessive positive charge may induce non‐specific interactions and increase the risk of cytotoxic effects.

Overall, TMC appears most suitable for mucosal and epithelial transport, thiolated chitosan for mucoadhesion and nucleic acid delivery, and CMC for biocompatible sustained release systems. Despite these advancements, direct head‐to‐head comparative studies remain limited, and variations in experimental conditions often complicate cross‐study interpretation. Therefore, standardized evaluation frameworks are needed to enable more reliable comparison and rational design of chitosan‐based vaccine systems.

### 2.5 Advantages of chitosan derivatives over traditional adjuvants

Chitosan derivatives offer several distinct advantages over conventional vaccine adjuvants such as aluminum salts and oil‐in‐water emulsions, particularly in the context of nanoparticle‐based vaccine delivery. Traditional adjuvants primarily function by enhancing humoral immune responses but often show limited ability to induce mucosal or cellular immunity. In contrast, chitosan derivatives exhibit multifunctional properties that enable both antigen delivery and immune stimulation. One of the key advantages of chitosan derivatives is their biodegradability and biocompatibility, which contribute to a favorable safety profile compared to conventional adjuvants that may cause local inflammation or reactogenicity. In addition, their cationic nature facilitates strong electrostatic interactions with negatively charged antigens, including proteins, peptides, DNA, and RNA, allowing efficient encapsulation and protection from enzymatic degradation. This feature is particularly important for next‐generation vaccines involving nucleic acids, where traditional adjuvants are largely ineffective.

Another significant advantage is their mucoadhesive property, which enables prolonged residence time at mucosal surfaces and enhances antigen uptake. This allows effective induction of mucosal immunity, including secretory IgA (sIgA) production, which is rarely achieved with conventional intramuscular adjuvants. Furthermore, chitosan derivatives can transiently open tight junctions between epithelial cells, thereby improving paracellular transport and antigen penetration. Chitosan derivatives also demonstrate the ability to induce a balanced immune response, activating both humoral and cellular pathways. They enhance antigen uptake by antigen‐presenting cells (APCs) and promote activation of T lymphocytes, which is critical for long‐term immunity and protection against intracellular pathogens. In contrast, traditional adjuvants such as alum are predominantly associated with Th2‐biased responses and limited cellular immunity. In addition, the physicochemical properties of chitosan derivatives can be tailored through chemical modification, allowing precise control over particle size, surface charge, antigen release profile, and targeting ability. This tunability provides a major advantage over conventional adjuvants, which generally lack structural flexibility.

Despite these advantages, it is important to note that challenges such as variability in degree of substitution, potential cytotoxicity at high charge density, and scalability considerations remain. Therefore, while chitosan derivatives represent a promising alternative to traditional adjuvants, further standardized comparative studies and clinical validation are required to fully establish their superiority. A detailed comparison between chitosan derivatives and traditional adjuvants is presented in Table 2.

**TABLE 2:** Comparison of chitosan derivatives with traditional vaccine adjuvants.

Parameter	Chitosan derivatives	Aluminum salts (alum)	Oil in water emulsions
Type of system	Polymeric nanoparticle based delivery and adjuvant system	Inorganic particulate adjuvant	Emulsion based adjuvant system
Biodegradability	Biodegradable and biocompatible	Non biodegradable	Biodegradable oils but may persist locally
Antigen encapsulation	Efficient encapsulation of proteins, peptides, DNA, RNA	Limited mainly to protein adsorption	Limited encapsulation, mainly antigen dispersion
Protection of antigen	Protects from enzymatic degradation	Minimal protection	Moderate protection
Immune response	Induces both humoral and cellular immunity	Primarily humoral (Th2 biased)	Mixed but mainly humoral
Mucosal immunity	Strong induction including IgA response	Poor or negligible	Limited mucosal response
Cellular uptake	Enhanced uptake via electrostatic interaction	Low uptake efficiency	Moderate uptake
Adjuvant mechanism	PRR activation, enhanced APC uptake, controlled release	Depot effect and inflammasome activation	Immune stimulation via antigen presentation enhancement
Surface charge	Positive (+20 to +50 mV typical)	Neutral to slightly negative	Neutral
Particle size	100–400 nm (tunable)	Micron sized aggregates	100–200 nm droplets
Controlled release	Yes, tunable release kinetics	Limited	Moderate
Suitability for nucleic acid vaccines	Highly suitable	Not suitable	Limited suitability
Mucoadhesion	Strong	Absent	Absent
Safety concerns	Possible cytotoxicity at high charge density	Local inflammation, granuloma formation	Reactogenicity, injection site reactions
Formulation flexibility	Highly tunable via chemical modification	Limited	Moderate
Clinical use status	Emerging and under investigation	Widely approved and used	Approved in selected vaccines

Abbreviations: APC, antigen‐presenting cell; PRR, pattern recognition receptor.

## 3 MECHANISMS OF IMMUNE ENHANCEMENT BY CHITOSAN DERIVATIVES

### 3.1 Interaction with antigen‐presenting cells

CDNPs play a pivotal role in enhancing vaccine‐induced immune responses by facilitating efficient interaction with APCs, particularly dendritic cells and macrophages (Figure 1). The cationic surface charge of CDNPs promotes strong electrostatic interactions with negatively charged cell membranes, leading to enhanced cellular adhesion and internalization through endocytic pathways. Following uptake, encapsulated antigens are released within endosomal or lysosomal compartments, where they undergo processing and are subsequently presented on major histocompatibility complex class I and II molecules. This antigen presentation initiates the activation of CD4^+^ helper T cells and CD8^+^ cytotoxic T lymphocytes, thereby bridging innate and adaptive immunity. In addition, CDNP‐mediated delivery protects antigens from premature degradation, ensuring efficient intracellular trafficking and sustained antigen availability for immune activation.^[13]^

**FIGURE 1:**
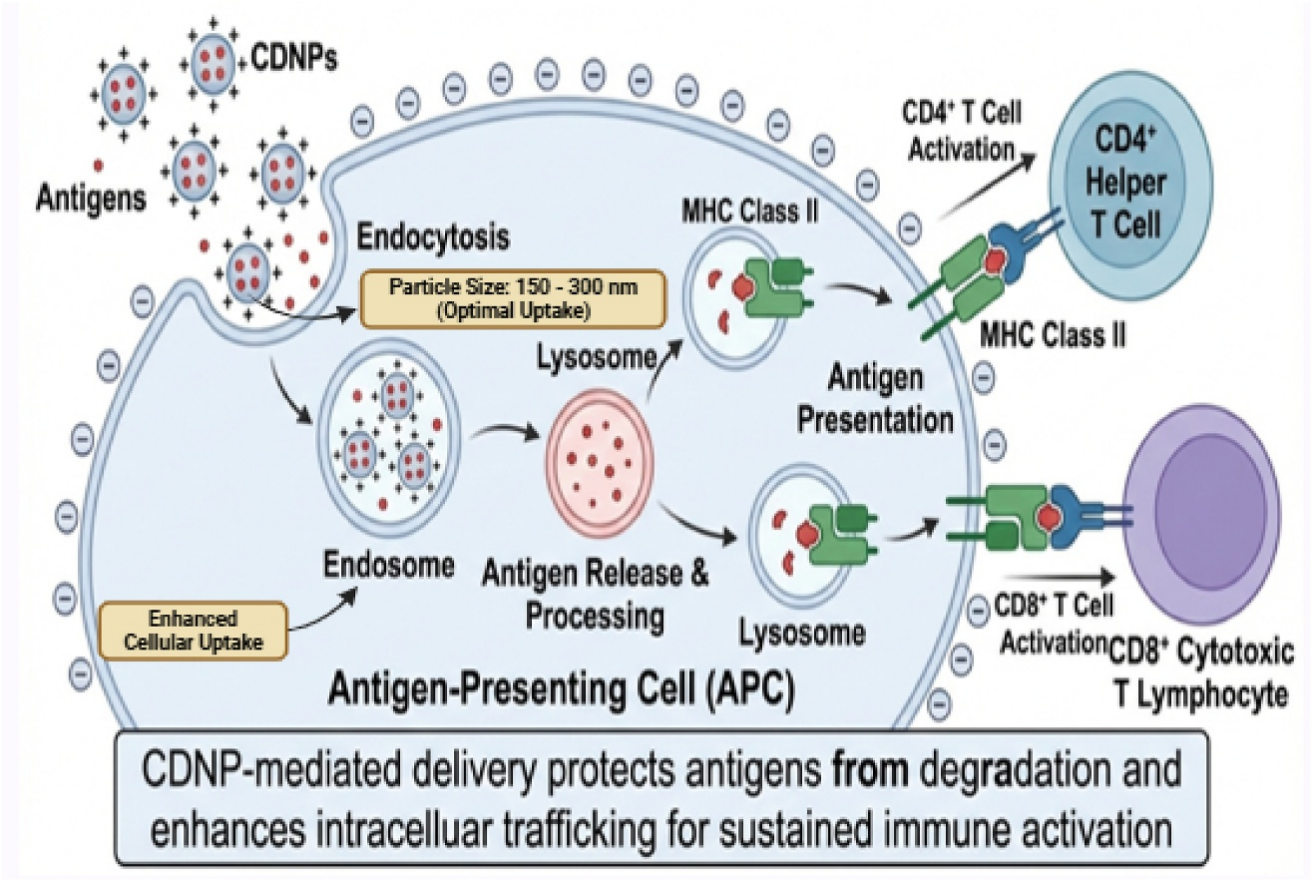
CDNP‐enhanced antigen presentation by APCs. APCs, antigen‐presenting cells; CDNP, chitosan derivative nanoparticle.

### 3.2 Modulation of cytokine profiles

Beyond facilitating antigen uptake, chitosan derivatives actively influence the cytokine microenvironment, thereby shaping the quality and magnitude of immune responses. As illustrated in Figure 2, CDNPs have been shown to stimulate the secretion of a broad spectrum of cytokines associated with both Th1‐type cellular immunity and Th2‐type humoral immunity, enabling a balanced immune response that is desirable for effective vaccination. For instance, derivatives such as TMC nanoparticles promote the production of pro‐inflammatory cytokines including IL‐2 and IFN‐γ, which support cytotoxic T‐cell activation, alongside Th2‐associated cytokines such as IL‐4 and IL‐10 that enhance antibody generation. This balanced cytokine modulation is particularly advantageous for subunit and nucleic acid vaccines, which often require immune amplification to achieve protective efficacy.^[14]^

**FIGURE 2:**
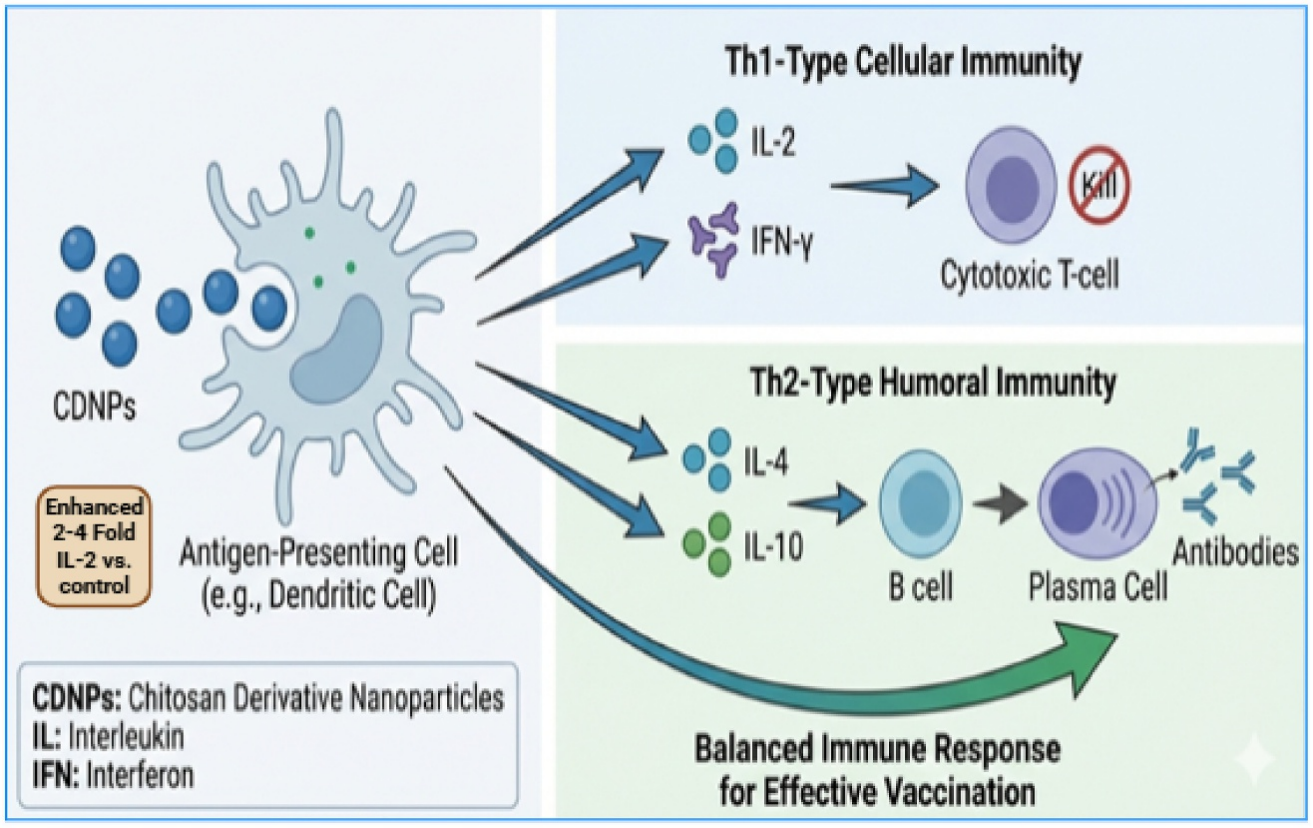
CDNPs mediated modulation of the cytokine microenvironment. CDNPs, chitosan derivative nanoparticles.

### 3.3 Induction of mucosal immunity

One of the most significant advantages of chitosan derivatives lies in their ability to induce robust mucosal immune responses, a feature often lacking in conventional adjuvants. The intrinsic mucoadhesive properties of CDNPs prolong their residence time at mucosal surfaces, such as the nasal, oral, and intestinal epithelium, thereby increasing the likelihood of antigen uptake. As illustrated in Figure 3, CDNPs facilitate transport of antigens across epithelial barriers and enhance uptake by specialized microfold (M) cells located in Peyer's patches and nasal‐associated lymphoid tissue. This targeted antigen delivery stimulates both local and systemic immune responses, leading to the production of sIgA antibodies, which play a critical role in neutralizing pathogens at their portals of entry in the respiratory and gastrointestinal tracts. Consequently, CDNP‐based vaccines are particularly promising for protection against mucosal pathogens.[^15^, ^16^]

**FIGURE 3:**
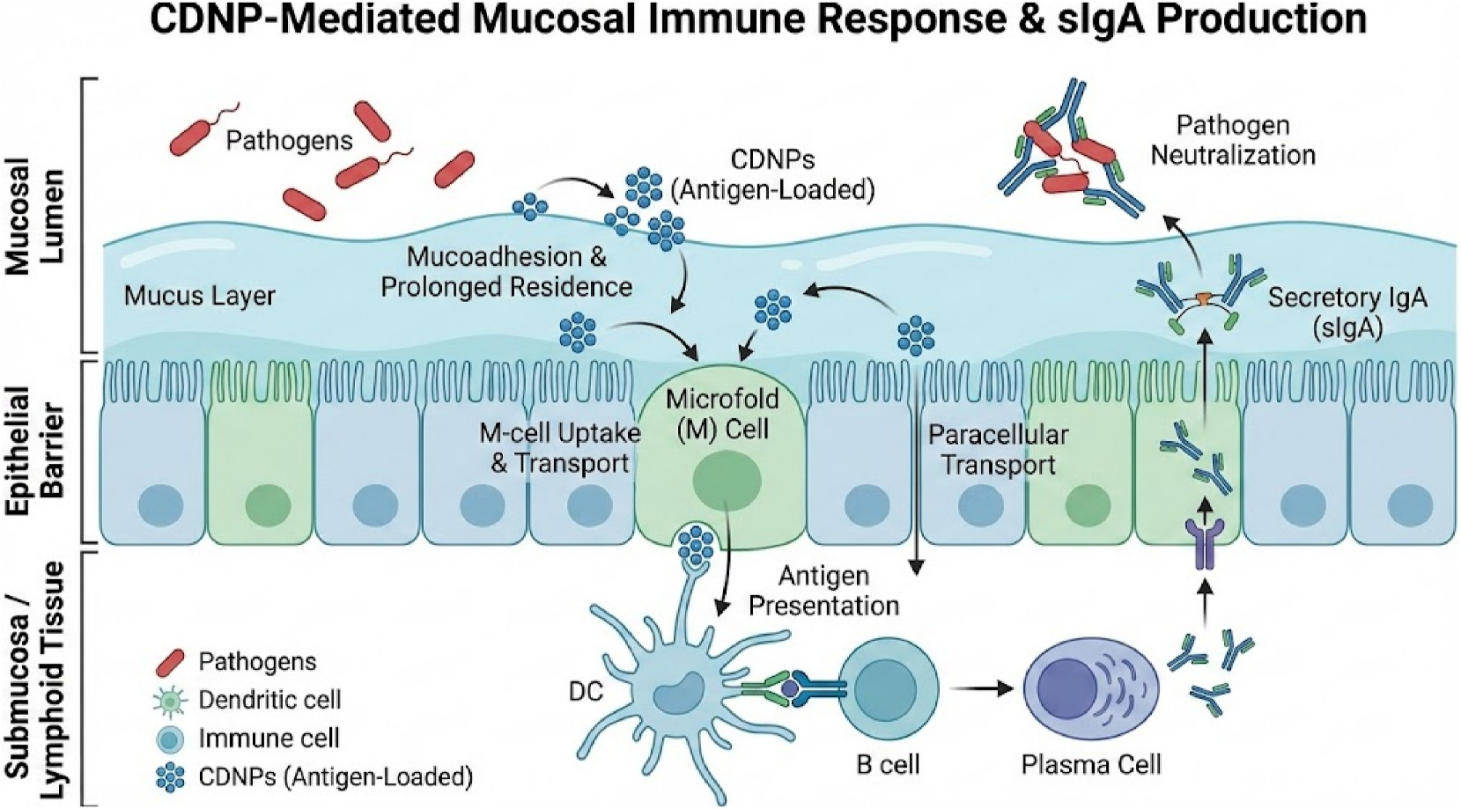
Schematic representation of the mechanism for CDNP‐mediated mucosal immune activation and (sIgA) production. CDNP, chitosan derivative nanoparticle; sIgA, secretory IgA.

### 3.4 Adjuvant and immunostimulatory effects

In addition to serving as antigen carriers, chitosan derivatives possess intrinsic adjuvant properties that enhance immune activation. As illustrated in Figure 4, CDNPs can stimulate innate immune pathways through interaction with pattern recognition receptors, including Toll‐like receptors expressed on APCs. Activation of these receptors initiates downstream signaling cascades that promote the maturation of dendritic cells, upregulation of costimulatory molecules, and secretion of inflammatory mediators. This innate immune activation provides critical signals required for the development of strong adaptive immune responses, including the differentiation of effector and memory B and T cells. As a result, chitosan derivatives function as multifunctional adjuvants, combining antigen delivery with immune potentiation, thereby improving vaccine efficacy and durability.^[17]^

**FIGURE 4:**
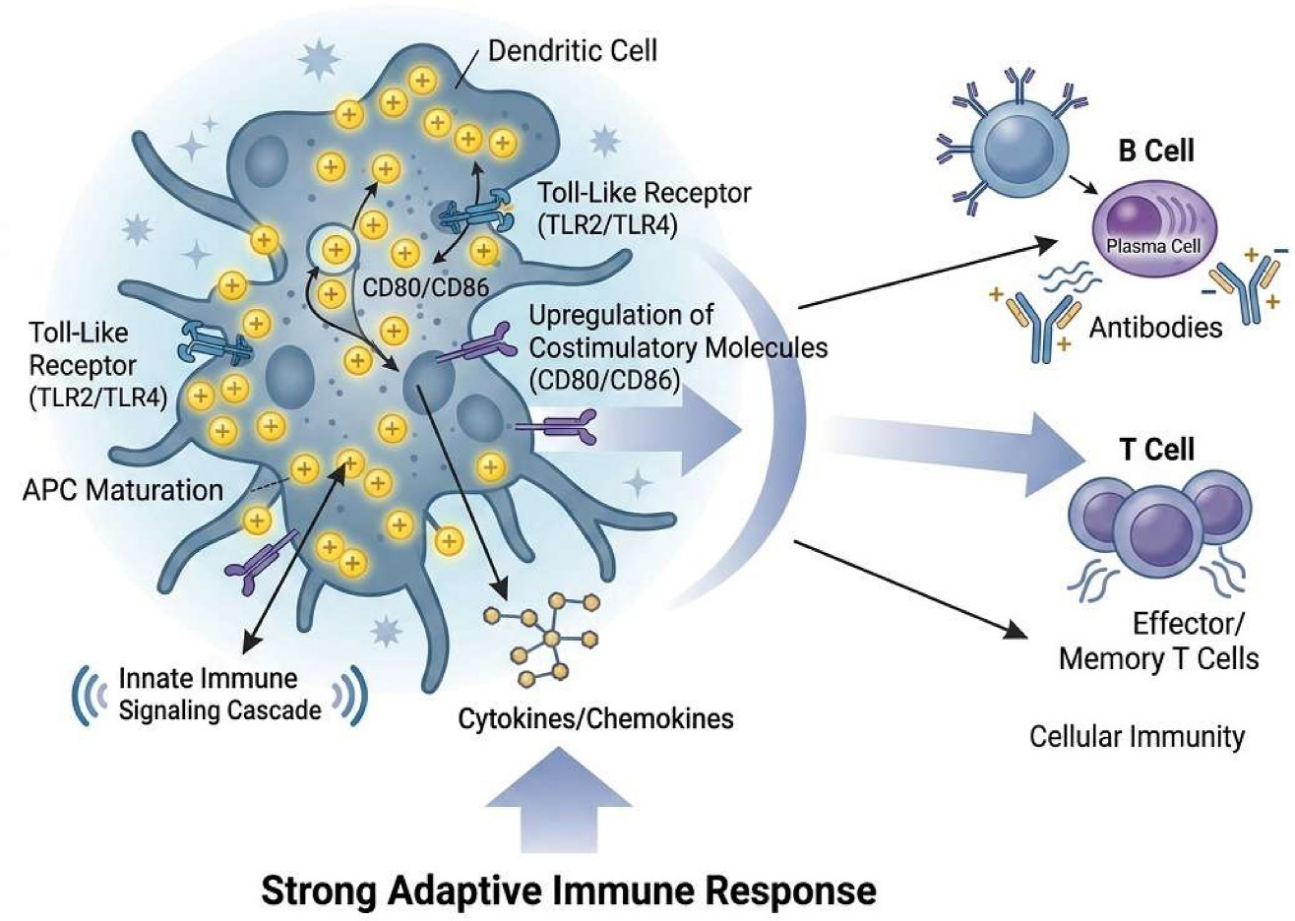
Mechanism of the intrinsic adjuvant and immunostimulatory effects of CDNPs. APC, antigen‐presenting cell; CDNPs, chitosan derivative nanoparticles; PRR, pattern recognition receptor.

## 4 PREPARATION TECHNIQUES OF CDNPs

The method of preparation plays a crucial role in determining the physicochemical properties, stability, antigen loading efficiency, and immunological performance of CDNPs. Several fabrication techniques have been developed to accommodate the diverse structural and biological requirements of vaccine antigens, particularly proteins and nucleic acids. Among these, ionic gelation, emulsion crosslinking, polyelectrolyte complexation, and drying‐based stabilization techniques are the most employed in vaccine delivery research.

### 4.1 Ionic gelation technique

Ionic gelation is one of the most widely used and well‐established methods for the preparation of CDNPs. This technique is based on the electrostatic interaction between positively charged chitosan derivatives and multivalent polyanions such as sodium tripolyphosphate (TPP). As illustrated in Figure 5, upon mixing aqueous solutions of chitosan derivatives and TPP under controlled conditions, spontaneous crosslinking occurs, leading to the formation of nanoparticles. The size and surface charge of the resulting nanoparticles can be finely tuned by adjusting parameters such as polymer concentration, pH, chitosan‐to‐TPP ratio, and stirring speed.

**FIGURE 5:**
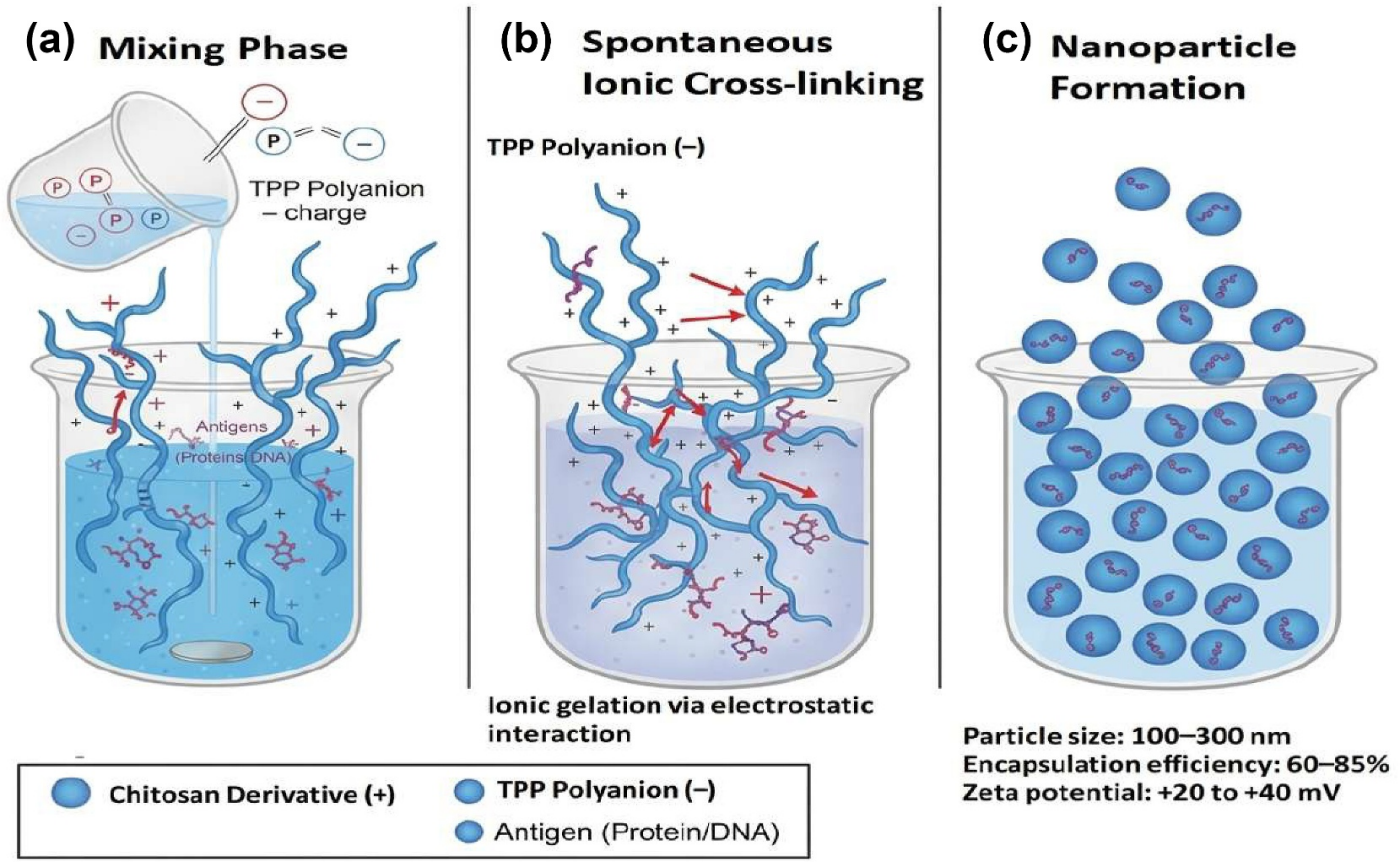
Ionic gelation method for chitosan derivative nanoparticle preparation.

A major advantage of ionic gelation is that it is conducted under mild, aqueous conditions without the use of organic solvents or high temperatures, making it particularly suitable for encapsulating labile vaccine antigens, including proteins, peptides, and nucleic acids. This method preserves antigen structural integrity and biological activity while providing effective encapsulation and sustained release. Consequently, ionic gelation has been extensively applied in the development of protein‐ and DNA‐based vaccines, where antigen stability is critical for eliciting effective immune responses.^[18]^ Additionally, the simplicity, reproducibility, and scalability of this technique make it attractive for both laboratory and industrial‐scale vaccine production.

### 4.2 Emulsion crosslinking method

The emulsion crosslinking method involves the formation of oil‐in‐water (O/W) or water‐in‐oil (W/O) emulsions, followed by chemical crosslinking of chitosan derivatives using agents such as glutaraldehyde. As illustrated in Figure 6, in this approach, chitosan derivative solutions containing the antigen are emulsified in an immiscible phase under high‐speed homogenization or sonication, leading to the formation of nanosized droplets. Subsequent crosslinking stabilizes these droplets, resulting in well‐defined nanoparticles with controlled morphology.

**FIGURE 6:**
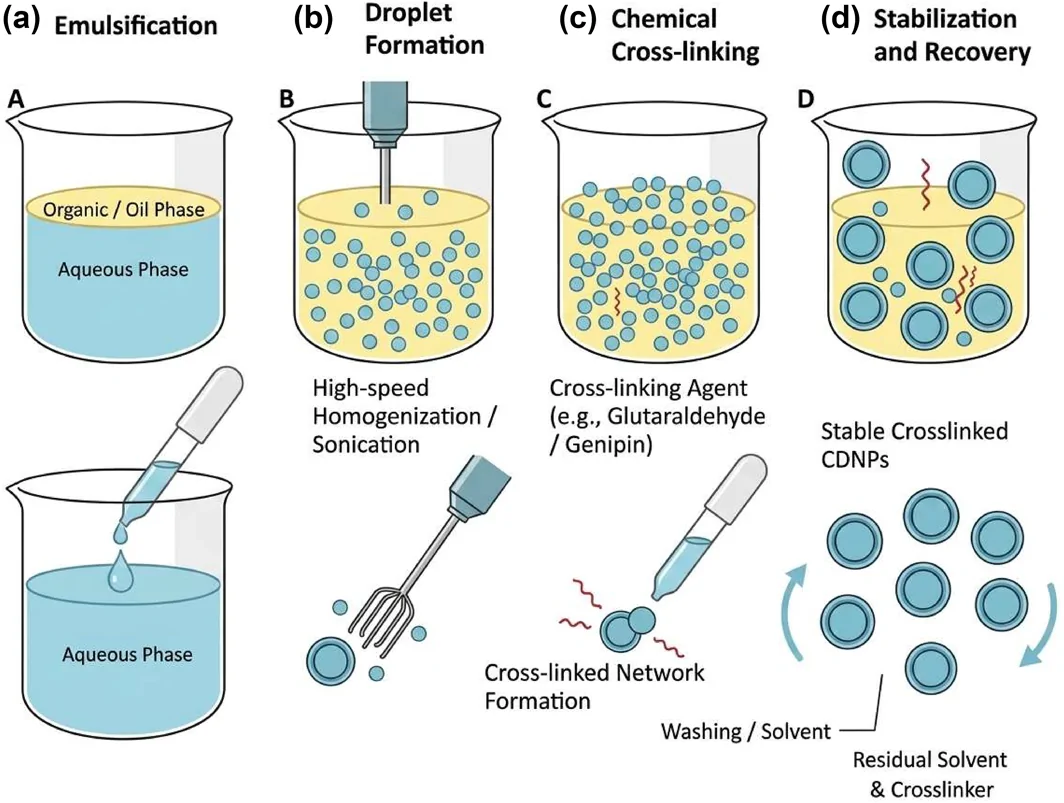
Schematic representation of the emulsion crosslinking method for chitosan derivative nanoparticles.

This technique offers the advantage of producing mechanically stable nanoparticles with narrow size distribution, which is particularly beneficial for achieving consistent antigen delivery and predictable release profiles. Emulsion crosslinking is useful when higher structural rigidity or prolonged release is required. However, the use of organic solvents and chemical crosslinking agents poses limitations as residual solvents or crosslinkers may compromise antigen stability or raise toxicity concerns. These factors necessitate extensive purification steps, which can increase process complexity and cost. As a result, while emulsion crosslinking remains valuable for certain applications, its use in vaccine delivery is generally restricted to antigens that can tolerate harsher processing conditions.^[19]^

### 4.3 Polyelectrolyte complexation

Polyelectrolyte complexation is a solvent‐free and chemically mild technique based on electrostatic interactions between positively charged chitosan derivatives and negatively charged antigens or polymers, such as DNA, RNA, or anionic proteins. Nanoparticles are formed spontaneously upon mixing the oppositely charged components in aqueous media, eliminating the need for crosslinking agents or organic solvents.

This approach is particularly advantageous for the delivery of nucleic acid vaccines, including DNA and mRNA, as it allows efficient complexation, protection from enzymatic degradation, and enhanced cellular uptake. Importantly, polyelectrolyte complexation preserves the biological activity of sensitive antigens and enables high encapsulation efficiency. The simplicity and versatility of this method make it highly attractive for next‐generation vaccine platforms, especially those targeting intracellular antigen expression and cellular immunity.^[20]^ Furthermore, the surface properties of nanoparticles produced by this method can be easily modified to enhance targeting or immunostimulatory effects.

### 4.4 Spray‐drying and freeze‐drying for nanoparticle stabilization

While nanoparticle fabrication methods focus on particle formation, post‐processing techniques such as spray‐drying and freeze‐drying (lyophilization) are essential for ensuring long‐term stability and practical applicability of CDNP‐based vaccines. These drying techniques convert nanoparticle suspensions into dry powders, which significantly enhance shelf‐life and facilitate storage, transportation, and administration.

Freeze‐drying is particularly advantageous for maintaining antigen integrity and nanoparticle structure, as it operates under low‐temperature conditions. The inclusion of cryoprotectants further minimizes aggregation and loss of bioactivity. Spray‐drying, on the other hand, enables rapid and scalable production of dry powders suitable for oral and nasal vaccine delivery, although careful optimization is required to prevent heat‐induced antigen degradation. Both techniques have been shown to preserve immunogenicity while improving formulation stability, thereby expanding the feasibility of CDNP‐based vaccines for mucosal immunization and global distribution.^[21]^

## 5 APPLICATIONS IN PRECLINICAL VACCINE STUDIES

CDNPs have been extensively investigated in preclinical models as versatile platforms for vaccine delivery. Their ability to encapsulate diverse antigens, protect them from degradation, and modulate immune responses has resulted in promising outcomes across protein‐, nucleic acid‐, mucosal‐, and cancer‐based vaccines. Numerous animal studies demonstrate that CDNP‐based formulations can induce robust systemic and mucosal immunity while maintaining favorable safety profiles.

### 5.1 Protein and peptide vaccines

Protein‐ and peptide‐based vaccines often require effective adjuvants and delivery systems to overcome their low intrinsic immunogenicity. Chitosan derivatives, particularly TMC and quaternized chitosan, have shown significant promise in this context. TMC nanoparticles loaded with influenza hemagglutinin antigen have been reported to induce strong antigen‐specific immune responses in murine models, characterized by elevated serum IgG levels as well as enhanced mucosal IgA production following intranasal administration. The improved immunogenicity is attributed to the enhanced solubility, mucoadhesion, and epithelial permeation properties of TMC, which facilitate antigen uptake and prolonged exposure to immune cells.^[22]^

Similarly, quaternized chitosan nanoparticles have been employed for the delivery of hepatitis B surface antigen (HBsAg), resulting in significantly increased antibody titers compared to conventional formulations. In addition to humoral immunity, these systems have been shown to stimulate cytotoxic T lymphocyte responses, indicating their capacity to promote cellular immunity, which is critical for long‐term protection. Such findings highlight the potential of chitosan derivatives to act not only as carriers but also as effective adjuvants for subunit vaccines.^[23]^

### 5.2 DNA and RNA vaccines

Nucleic acid–based vaccines, including DNA and RNA vaccines, offer several advantages such as rapid development and the ability to induce both humoral and cellular immunity; however, their clinical translation is often hindered by instability and poor cellular uptake. CDNPs provide an effective solution by protecting nucleic acids from enzymatic degradation and facilitating efficient delivery into target cells. The cationic nature of chitosan derivatives enables strong electrostatic complexation with negatively charged DNA or RNA, resulting in enhanced stability and transfection efficiency.

Among various derivatives, thiolated chitosan nanoparticles have demonstrated superior performance in DNA vaccine delivery. Preclinical studies have shown that plasmid DNA encapsulated in thiolated chitosan nanoparticles leads to enhanced gene expression, robust antigen‐specific IgG production, and strong T‐cell responses in animal models. The improved efficacy is largely attributed to increased cellular uptake, prolonged intracellular retention, and controlled antigen expression, which together promote effective immune priming.^[24]^ These findings underscore the suitability of CDNPs for next‐generation DNA and mRNA vaccine platforms.

### 5.3 Mucosal vaccines

Mucosal vaccination is an attractive strategy for preventing infections caused by pathogens that enter the body through respiratory or gastrointestinal routes. Chitosan derivatives are particularly well suited for this application due to their mucoadhesive properties and ability to enhance epithelial permeability. Preclinical studies have demonstrated that intranasal administration of CDNP‐based vaccines against respiratory pathogens, including influenza viruses and SARS‐CoV‐2, elicits strong mucosal immune responses, as evidenced by high levels of sIgA in nasal secretions. In addition, these formulations induce systemic immunity, providing a dual layer of protection.^[25]^

Oral vaccination using CDNP‐encapsulated antigens has also shown promising results in animal models. The nanoparticles protect antigens from harsh gastrointestinal conditions and facilitate uptake by M cells in Peyer's patches, leading to the activation of gut‐associated lymphoid tissue. Such formulations have been reported to induce antigen‐specific IgA responses in the intestinal mucosa, along with systemic antibody production, making them attractive candidates for vaccines targeting enteric pathogens.^[26]^ Collectively, these studies highlight the potential of CDNPs to overcome key challenges associated with mucosal vaccine delivery.

### 5.4 Immunotherapy and cancer vaccines

Beyond prophylactic vaccines, CDNPs have been explored in therapeutic cancer vaccines and immunotherapy applications. CDNPs have been employed to deliver tumor‐associated antigens, leading to enhanced antigen presentation and activation of tumor‐specific T‐cell responses in murine models. These immune responses have been associated with significant tumor growth inhibition and, in some cases, tumor regression, demonstrating the therapeutic potential of CDNP‐based cancer vaccines.^[27]^

Furthermore, the co‐delivery of tumor antigens with immunomodulatory agents, such as cytokines or TLR agonists, using CDNPs has been shown to further enhance antitumor efficacy. This combinatorial approach amplifies immune activation by simultaneously promoting antigen presentation and innate immune stimulation, resulting in stronger and more durable antitumor responses. Such findings position CDNPs as promising multifunctional platforms for cancer immunotherapy and personalized vaccine strategies.^[28]^

## 6 CLINICAL STUDIES AND TRANSLATIONAL POTENTIAL

Although the majority of research on CDNPs remains at the preclinical stage, accumulating evidence from early‐phase clinical studies highlights their translational promise as vaccine adjuvants and delivery systems. The favorable safety profile, biodegradability, and immunostimulatory properties of chitosan derivatives have facilitated their evaluation in human subjects, particularly for mucosal and subunit vaccines.

Clinical investigations of influenza vaccines employing TMC nanoparticles have demonstrated encouraging outcomes. Intranasal administration of TMC‐based formulations in human volunteers was shown to be well tolerated, with no significant adverse effects reported. Importantly, these formulations induced robust mucosal immune responses, as evidenced by increased levels of sIgA in nasal secretions, along with measurable systemic antibody responses. These findings underscore the potential of chitosan derivatives to overcome the limitations of conventional intramuscular vaccines by effectively inducing mucosal immunity, which is critical for protection against respiratory pathogens.^[29]^

Similarly, hepatitis B vaccines formulated with CDNPs have progressed into early clinical evaluation. CDNP‐based formulations were found to enhance antigen‐specific antibody responses compared with conventional alum‐adjuvanted vaccines. Notably, these systems enabled dose‐sparing strategies, where reduced antigen doses elicited comparable or improved immunogenicity. Such an approach is particularly advantageous for large‐scale immunization programs, as it can reduce vaccine costs and improve global accessibility without compromising efficacy.^[30]^

In the context of COVID‐19 and other emerging infectious diseases, chitosan‐based nanoparticle platforms have attracted growing interest as delivery vehicles for nucleic acid vaccines, including mRNA formulations. Early‐stage clinical and translational studies are currently exploring the ability of chitosan derivatives to improve mRNA stability, enhance cellular uptake, and modulate immune responses while maintaining an acceptable safety profile. Although these investigations are still in preliminary phases, they highlight the adaptability of CDNPs for next‐generation vaccine technologies and their potential role in rapid‐response vaccination strategies against emerging pathogens.^[31]^

Collectively, these clinical findings suggest that CDNPs represent a promising and versatile platform capable of bridging the gap between experimental vaccine formulations and clinically viable products. Continued clinical evaluation, coupled with advances in scalable manufacturing and regulatory standardization, will be essential for the successful translation of CDNP‐based vaccines into widespread clinical use.

## 7 CHALLENGES AND FUTURE PERSPECTIVES

Despite extensive research, a major limitation in the current literature is the lack of standardized comparative studies evaluating different chitosan derivatives under identical experimental conditions. Most studies report outcomes using varying formulations, antigen types, and evaluation models, making direct performance comparison difficult. This inconsistency limits the ability to identify the most effective derivative for specific vaccine applications and highlights the need for systematic, head‐to‐head investigations.^[32]^

### 7.1 Stability and scalability

One of the major challenges associated with CDNP‐based vaccines is ensuring long‐term physicochemical stability, particularly during storage and transportation. Nanoparticle aggregation, changes in particle size distribution, and loss of surface charge can adversely affect antigen loading efficiency, release kinetics, and immunogenicity. Additionally, antigen degradation during formulation or storage remains a concern, especially for sensitive biomolecules such as proteins and nucleic acids.^[33]^

To address these issues, optimization of lyophilization (freeze‐drying) and spray‐drying techniques has gained increasing attention. The use of appropriate cryoprotectants and stabilizers can preserve the nanoparticle integrity and antigen bioactivity during drying and reconstitution. Furthermore, scalable and reproducible manufacturing processes must be established to ensure batch‐to‐batch consistency, a prerequisite for industrial‐scale vaccine production. Advances in continuous manufacturing and process analytical technologies may play a pivotal role in overcoming scalability limitations.

### 7.2 Regulatory considerations

Regulatory approval represents a significant hurdle for the clinical adoption of CDNP‐based vaccines, particularly because chitosan derivatives are considered novel adjuvants and delivery systems. Regulatory agencies require comprehensive evaluation of nanoparticle‐based formulations, including detailed characterization of particle size, surface charge, morphology, encapsulation efficiency, and release profiles.

The absence of universally accepted standardized characterization protocols for polymeric nanoparticle vaccines complicates regulatory assessment. Moreover, demonstrating consistent quality control across large‐scale production batches is critical for meeting regulatory expectations. Clear regulatory guidelines specific to nanotechnology‐based vaccines are still evolving, which may prolong approval timelines. Therefore, early engagement with regulatory authorities and the development of harmonized testing frameworks will be essential to streamline clinical translation.

### 7.3 Immunogenicity and safety

Although chitosan derivatives are generally regarded as safe and well tolerated, careful evaluation of their immunogenicity and safety profiles remains essential. Excessive immune stimulation or unintended systemic inflammatory responses could compromise vaccine safety, particularly with repeated dosing or high antigen loads. The cationic nature of chitosan derivatives, while beneficial for antigen delivery, may also contribute to non‐specific immune activation if not carefully controlled.

Comprehensive dose‐optimization studies are required to balance immunogenic efficacy with safety. Additionally, long‐term studies assessing repeated administration, biodistribution, and potential accumulation of nanoparticles are necessary to fully understand chronic exposure risks. Such investigations are particularly important for vaccines intended for pediatric populations or for repeated booster immunizations.

### 7.4 Future directions

Looking ahead, several strategies hold promise for enhancing the performance and versatility of CDNP‐based vaccines. Combination approaches, involving the co‐delivery of chitosan derivatives with TLR agonists or other immunostimulatory molecules, may result in synergistic immune activation and improved vaccine efficacy. These multifunctional systems can simultaneously enhance antigen presentation and innate immune signaling.

Another promising direction is the development of targeted vaccine delivery systems, achieved through surface functionalization of CDNPs with ligands specific to dendritic cells, macrophages, or mucosal epithelial cells. Such targeting strategies can improve antigen localization, reduce off‐target effects, and enhance immune specificity.

Finally, CDNPs are well‐positioned to support next‐generation vaccine technologies, including mRNA, DNA, and recombinant subunit vaccines for emerging and re‐emerging infectious diseases. Their adaptability, safety, and ability to induce both systemic and mucosal immunity make them attractive candidates for rapid‐response vaccine platforms. Continued interdisciplinary research integrating immunology, materials science, and pharmaceutical technology will be crucial for realizing the full potential of CDNPs in future vaccine development.^[34]^

## 8 CONCLUSION

Chitosan derivatives have emerged as a versatile and highly promising platform for nanoparticle‐based vaccine delivery, offering a unique combination of antigen protection, mucoadhesive properties, and intrinsic immunostimulatory activity. Their cationic nature and structural tunability enable efficient encapsulation of a wide range of antigens, including proteins, peptides, and nucleic acids, while facilitating enhanced uptake by APCs and prolonged residence at mucosal surfaces. These characteristics collectively contribute to the induction of robust and balanced immune responses encompassing both systemic humoral and cellular immunity, as well as mucosal immune responses critical for protection against pathogens entering through epithelial barriers.

Extensive preclinical investigations, supported by emerging clinical evidence, demonstrate that CDNP‐based vaccines can significantly enhance immunogenicity while maintaining a favorable safety profile, attributable to their biodegradability and low toxicity. Their adaptability to diverse routes of administration, particularly intranasal and oral delivery, further underscores their potential as next‐generation vaccine adjuvants and delivery systems.

Nevertheless, challenges related to formulation stability, large‐scale manufacturing, and regulatory standardization remain key obstacles to widespread clinical translation. Addressing these limitations through advanced formulation strategies, scalable manufacturing technologies, and harmonized regulatory frameworks will be critical for future success. With continued interdisciplinary research and rational design, CDNP‐based vaccine platforms hold substantial promise to transform vaccine development, particularly in the context of mucosal immunization, emerging infectious diseases, and modern nucleic acid‐based immunotherapies.

## CONFLICT OF INTEREST STATEMENT

The authors declare no conflicts of interest.

## Data Availability

The data that support the findings of this study are available from the corresponding author upon reasonable request.
